# Do salivary bypass tubes lower the incidence of pharyngocutaneous fistula following total laryngectomy? A retrospective analysis of predictive factors using multivariate analysis

**DOI:** 10.1007/s00405-016-4391-9

**Published:** 2016-12-23

**Authors:** Robert W. A. Hone, Eqramur Rahman, Gentle Wong, Yvette Annan, Victoria Alexander, Ali Al-Lami, Kiran Varadharajan, Michael Parker, Ricard Simo, Lisa Pitkin, Alasdair Mace, Enyinnaya Ofo, Alistair Balfour, Iain J. Nixon

**Affiliations:** 1 Faculty of Medical Science, Post Graduate Medical Institute, Anglia Ruskin University, Bishop Hall Lane, Chelmsford, Essex UK; 2 Ear, Nose and Throat Department, Brighton and Sussex University Hospital, 177 Preston Rd, Brighton, UK; 3 Ear, Nose and Throat Department, Charing Cross Hospital, Fulham Palace Rd, London, UK; 4 Ear, Nose and Throat Department, Guy’s Hospital, Great Maze Pond, London, UK; 5 Ear, Nose and Throat Department, St Georges Hospital, Blackshaw Rd, London, UK; 6 Ear, Nose and Throat Department, William Harvey Hospital, Kennington Road, Willesborough, Ashford, Kent UK; 7 Ear, Nose and Throat Department, Royal Surrey County Hospital, Egerton Rd, Guildford, Surrey UK; 8 Medway Maritime Hospital, Windmill Road, Gillingham, Kent ME7 5NY UK

**Keywords:** Fistula, Laryngectomy, Cancer, Complications, Salivary bypass tube

## Abstract

Salivary bypass tubes (SBT) are increasingly used to prevent pharyngocutaneous fistula (PCF) following laryngectomy and pharyngolaryngectomy. There is minimal evidence as to their efficacy and literature is limited. The aim of the study was to determine if SBT prevent PCF. The study was a multicentre retrospective case control series (level of evidence 3b). Patients who underwent laryngectomy or pharyngolaryngectomy for cancer or following cancer treatment between 2011 and 2014 were included in the study. The primary outcome was development of a PCF. Other variables recorded were age, sex, prior radiotherapy or chemoradiotherapy, prior tracheostomy, type of procedure, concurrent neck dissection, use of flap reconstruction, use of prophylactic antibiotics, the suture material used for the anastomosis, tumour T stage, histological margins, day one post-operative haemoglobin and whether a salivary bypass tube was used. Univariate and multivariate analysis were performed. A total of 199 patients were included and 24 received salivary bypass tubes. Fistula rates were 8.3% in the SBT group (2/24) and 24.6% in the control group (43/175). This was not statistically significant on univariate (*p* value 0.115) or multivariate analysis (*p* value 0.076). In addition, no other co-variables were found to be significant. No group has proven a benefit of salivary bypass tubes on multivariate analysis. The study was limited by a small case group, variations in tube duration and subjects given a tube may have been identified as high risk of fistula. Further prospective studies are warranted prior to recommendation of salivary bypass tubes following laryngectomy.

## Introduction

Pharyngocutaneous fistulae (PCF) are the most common serious complication following total laryngectomy or pharyngolaryngectomy [1, 2]. The incidence of PCF is reported to be between 3 and 65% [3–8] and they have been estimated to cost one Canadian centre around $400,000 a year [5].

Some units now advocate the use of salivary bypass tubes (SBT) following laryngectomy to minimise the chance of developing a PCF. However, the evidence base for the use of SBT is limited. Literature is descriptive with the exception of three studies. Two are retrospective single centre studies where univariate analysis was used to determine an association between PCF and SBTs [9, 10]. The third, a multicentre retrospective study suggested a benefit of salivary bypass tubes on univariate analysis, but lacked statistical power for multivariate analysis, and therefore, failed to show an independent effect [11].

Given the high potential for morbidity and even mortality related to PCF, all efforts should be taken to prevent this complication. The aim of this study was to analyse factors associated with PCF following laryngectomy or pharyngolaryngectomy in a cohort large enough to allow multivariate analysis.

## Materials and methods

The study was performed as part of a Master’s degree at the Anglia Ruskin University. It is a retrospective case control analysis. Patients who underwent a laryngectomy or pharyngolaryngectomy following diagnosis or treatment of laryngeal cancer or pharyngeal cancer involving the larynx from the 1st of January 2011 until the 31st of December 2014 were included in the study. The University Faculty Research Ethics Panel was consulted and approval for the study was obtained provided individual NHS trust research and development (R&D) departments gave approval. Seven head and neck departments in the South East of England were invited to participate and all agreed to contribute. Research Ethics Committee Proportionate Review was granted via the online Integrated Research Applications System and individual NHS trust R&D departments were contacted to secure relevant permissions which were all obtained.

Patients were identified through head and neck multidisciplinary team meetings and theatre logbooks. Patient notes were reviewed to collect data including age, sex, prior radiotherapy or chemoradiotherapy, prior tracheostomy, type of procedure (laryngectomy or pharyngolaryngectomy), concurrent neck dissection (unilateral or bilateral), use of flap reconstruction, use of prophylactic antibiotics (for 72 h or more), suture material used for the anastomosis, tumour T stage (as per histological specimen during the resection except for patients who received a laryngectomy for a dysfunctional larynx where the original tumour T stage was used and this was recorded from imaging and clinical examination findings), histological margins (R0, R1 or R2), day one post-operative haemoglobin, whether a salivary bypass tube was used and whether the patient developed a PCF.

Data was analysed using univariate and multivariate analysis on the computer programme R from the R Foundation for Statistical Computing. Binary and nominal data was analysed using the Fisher’s exact Test to generate *p* values for potential predictors of post-operative fistula independently of the influence of co-variables. Continuous variables were analysed with permutation tests using the Monte Carlo algorithm. Standard errors and 95% Confidence intervals were generated by bootstrap methodology. Standard deviations are based on group data rather than pooled estimates; however, standard errors of differences between 95% confidence intervals were generated with 9999 bootstrap samples and *p* values obtained with 10,000 permutation samples. The type of flap was excluded from statistical analysis due to the relatively small numbers in the large number of different groups and potential combinations of the variables.

Multivariate analysis is a correlation study and aims to assess the relationship between variables. It relies on the sample being random; otherwise there is a risk of bias. The relationship between the presence and absence of post-operative fistula was assessed using multiple predictor binary logistical regression models with 13 variables as potential predictive factors. Multivariate logistic models were fitted with an algorithm package followed by a predictor selection algorithm. The model was then fitted with penalised maximum likelihood estimations. A Receiver-Operator Characteristic (ROC) curve was generated to assess whether the model is a good predictor of the occurrence of post-operative fistulae.

## Results

(i) Summary of data.

All head and neck centres invited to take part provided data for the study. A total of 271 patients were initially identified. There were 72 exclusions resulting in a total of 199 patients used for statistical analysis. Reasons for exclusions included incomplete data and missing notes (44 patients), incorrectly coded patients who had different surgery (23 patients), patients having a laryngectomy without a history of malignancy (2 patients) and patients from the incorrect time period (3 patients). Five laryngectomies were performed for a dysfunctional larynx following previous radiotherapy or chemoradiotherapy. For the purposes of analysis these patients were assigned their original T stage (one T1, three T2 and one T3 tumour).

The median patient age was 66 years (range 36–89 years). There were 32 (16%) female and 167 (86%) male subjects. A total of 142 (71%) patients had a laryngectomy while a further 57 (29%) underwent pharyngolaryngectomy. Previous radiotherapy or chemoradiotherapy had been performed in 96 patients meaning 48% of patients were having salvage surgery with 103 (52%) patients undergoing primary surgery. Pre-operative tracheostomy was performed in 43 (22%) patients. A total of 147 (74%) patients underwent concurrent neck dissection. Post-operative haemoglobin ranged from 5.3 to 14.6 g/dl with a median of 10.0 g/dl. Vicryl was the most commonly used suture type in 175 (88%) procedures. Alternative suture material included vicryl rapide in three patients (2%), polydioxanone (PDS) in 17 patients (9%) and staples in four patients (2%). Tumour T stage ranged from T1 to T4 with 10 (5%) T1 tumours, 14 (7%) T2 tumours, 44 (22%) T3 tumours and 131 (66%) T4 tumours (T4a and T4b subgroups were combined). Histological margins were positive (R1 or R2) in 42 (21%) patients and negative (R0 or negative due to no malignant disease) in the remaining 157 (79%) patients. Post-operative antibiotics were given in 134 (67.3%) cases and reconstructive flaps were used in 62 (31%). Reconstructive flaps used included 31 (50%) pectoralis major, 15 (24%) anterolateral thigh flap (ALT), 8 (13%) jejunal free flaps, five (8%) radial forearm free flaps and two (2%) had a combination of a pectoralis major flap with a free flap. Twenty-four patients (12%) had salivary bypass tube inserted during surgery.

A total of 45 fistulae developed in the early post-operative period, an incidence of 23%. However, in the group treated with a SBT only two post-operative fistulae developed (8%) compared to 26% in the control group. Table 1 shows the co-variables in the primary outcome group (patients who received a salivary bypass tube) compared to the control group. The descriptive statistics of the group as a whole and stratified by the use of a SBT are also shown in Table 1.

**Table 1:** A summary of co-variables in the salivary bypass group compared to the control group cohort demographics, and fistula incidence with univariate statistical analysis

Co-variant	Number of patients	Salivary Bypass Tube Group	No Salivary Bypass Tube	Post-operative fistula	No post-operative fistula	Fisher exact test (*p* value)
	*n* = 199	*n* = 24	*n* = 175	*n* = 45	*n* = 154	
Sex						
Male	167 (83.9%)	21 (87.5%)	146 (83.4%)	37 (82.2%)	130 (84.4%)	0.818
Female	32 (16.1%)	3 (12.5%)	29 (16.6%)	8 (17.8%)	24 (15.6%)	
Type of surgery						
Laryngectomy	142 (71.4%)	16 (66.7%)	126 (72.0%)	35 (77.8%)	107 (69.5%)	0.350
Pharyngolaryngectomy	57 (28.6%)	8 (33.3%)	49 (28.0%)	10 (22.2%)	47 (30.5%)	
Radiotherapy or chemoradiotherapy						
Yes	96 (48.2%)	13 (54.2%)	83 (47.4%)	20 (44.4%)	76 (49.4%)	0.613
No	103 (51.8%)	11 (45.8%)	92 (52.6%)	25 (65.6%)	78 (50.6%)	
Previous tracheostomy						
Yes	43 (21.6%)	0 (0%)	43 (24.6%)	11 (24.4%)	32 (20.8%)	0.681
No	156 (78.4%)	24 (100%)	132 (75.4%)	34 (75.6%)	122 (79.2%)	
Tumour T stage						
T1	10 (5.0%)	2 (8.3%)	8 (4.6%)	3 (6.7%)	7 (4.5%)	0.185
T2	14 (7.0%)	3 (12.5%)	11 (6.3%)	6 (13.3%)	8 (5.2%)	
T3	44 (22.1%)	6 (25%)	38 (21.7%)	7 (15.6%)	37 (24.0%)	
T4	131 (65.8%)	13 (54.2%)	118 (67.4%)	29 (64.4%)	102 (66.2%)	
Positive margins on histology						
Yes	42 (21.1%)	5 (20.8%)	37 (21.1%)	10 (22.2%)	32 (20.8%)	0.837
No	157 (78.9%)	19 (78.2%)	138 (78.9%)	35 (77.8%)	122 (79.2%)	
Concurrent neck dissection						
Yes	147 (73.9%)	16 (66.7%)	131 (74.9%)	30 (66.7%)	117 (76%)	0.248
No	52 (26.1%)	8 (33.3%)	44 (25.1%)	15 (33.3%)	37 (24.0%)	
Prophylactic antibiotics						
Yes	134 (67.3%)	17 (70.8%)	117 (66.9%)	27 (60%)	107 (69.5%)	0.279
No	65 (32.7%)	7 (29.2%)	58 (33.1%)	18 (40%)	47 (30.5%)	
Reconstructive flap						
Yes	62 (31.2%)	13 (54.2%)	49 (28.0%)	15 (33.3%)	47 (30.5%)	0.718
No	137 (68.8%)	11 (45.8%)	126 (72.0%)	30 (66.7%)	107 (69.5%)	
Salivary bypass tube inserted						
Yes	24 (12.1%)	24 (100%)	0 (0%)	2 (4.4%)	22 (14.3%)	0.115
No	175 (87.9%)	0 (0%)	175 (100%)	43 (95.6%)	132 (85.7%)	
Age range, years	36–89	36–86	41–89	45–89	36–88	–
Post-operative haemoglobin range, g/dl	5.3–14.6	7.1–13.9	5.3–14.6	7.4–13.0	5.3–14.6	–
Fistula developed						
Yes	45 (22.6%)	2 (8.3%)	43 (24.6%)	45 (22.6%)	154 (77.4%)	–
No	154 (77.4%)	22 (91.7%)	132 (75.4%)			–

(ii) Univariate analysis.

No individual risk factor was associated with post-operative fistula. No *p* value from Fisher’s exact test or permutation testing reached statistical significance of less than 0.05. The *p* values for Fisher’s exact test are summarised in Table 1 and the values from the quantitative variables which underwent permutation testing to generate *p* values and the results are summarised in Table 2.

**Table 2:** Univariate statistical analysis of continuous variables using 10,000 permutations and 9999 bootstrap calculations

Continuous variables	Range	Median	Mean	Standard deviation	Standard error	95% confidence limits		*p* value
						Lower	Upper	
Age (years)								
Yes	45–89	67	66.2	10.49	10.50 (59.0–74.0) [45.0–89.0]	−3.7	3.0	0.877
No	36–88	66	65.9	9.78				
Total	36–89	66	66.0	9.91				
Post-operative haemoglobin (g/dl)								
Yes	7.4–13.0	10	10.02	1.400	1.400 (8.9–11.1) [7.4–13.0]	−0.06	0.93	0.142
No	5.3–14.6	10.2	10.45	1.784				
Total	5.3–14.6	10.2	10.36	1.712				

The salivary bypass tube groups were compared to see if the patients receiving SBT were comparable using Fisher’s exact test and permutation testing. The proportion of patients who had a salivary bypass tube were significantly more likely to have had a flap reconstruction (*p* = 0.017) and significantly less likely to have had a tracheostomy (*p* = 0.003). All other variables were found to be insignificant.

(iii) Multivariate analysis.

The predictors collected were analysed in the search algorithm and the results of the binary logistic regression analysis are shown in Table 3 and the results using multiple-predictor binary logistical regression with fitted penalised maximum likelihood estimations are shown in Table 4.

**Table 3:** Binary logistic regression model results of response variable presence of post-operative fistulae related to single predictors

Predictor	Category	*n*	Presence of post-operative fistulae		Odds ratio (95% confidence limits)	*p* value
			No, *n* = 154	Yes, *n* = 45		
Sex	Overall	199	154 (77.4%)	45 (22.6%)		0.671
	Female	32	24 (75.0%)	8 (25.0%)	1.000	
	Male	167	130 (77.8%)	37 (22.2%)	0.828 (0.361, 2.056)	0.671
Age at admission (years)	_	199			1.003 (0.970, 1.037)	0.860
Laryngectomy or pharyngolaryngectomy	Overall	199	154 (77.4%)	45 (22.6%)		0.296
	Laryngectomy	142	107 (75.4%)	35 (24.6%)	1.000	
	Pharyngolaryngectomy	57	47 (82.5%)	10 (17.5%)	0.669 (0.298, 1.405)	0.296
Previous RT or chemo RT	Overall	199	154 (77.4%)	45 (22.6%)		0.568
	No	103	78 (75.7%)	25 (24.3%)	1.000	
	Yes	96	76 (79.2%)	20 (20.8%)	0.825 (0.423, 1.595)	0.568
Previous tracheostomy	Overall	199	154 (77.4%)	45 (22.6%)		0.566
	No	156	122 (78.2%)	34 (21.8%)	1.000	
	Yes	43	32 (74.4%)	11 (25.6%)	1.256 (0.563, 2.667)	0.566
Concurrent neck dissection	Overall	199	154 (77.4%)	45 (22.6%)		0.207
	No	52	37 (71.2%)	15 (28.8%)	1.000	
	Yes	147	117 (79.6%)	30 (20.4%)	0.628 (0.310, 1.302)	0.207
Post-operative Hb (g/dl)	_	199			0.862 (0.700, 1.049)	0.141
Suture type used for anastomosis	Overall	199	154 (77.4%)	45 (22.6%)		0.371
	PDS	17	13 (76.5%)	4 (23.5%)	1.000	
	Rapide	3	1 (33.3%)	2 (66.7%)	5.000 (0.526, 66.878)	0.159
	Staples	4	3 (75.0%)	1 (25.0%)	1.286 (0.104, 10.799)	0.824
	Vicryl	175	137 (78.3%)	38 (21.7%)	0.840 (0.290, 2.896)	0.764
Tumour T stage	Overall	199	154 (77.4%)	45 (22.6%)		0.204
	T1	10	7 (70.0%)	3 (30.0%)	1.000	
	T2	14	8 (57.1%)	6 (42.9%)	1.639 (0.329, 9.096)	0.549
	T3	44	37 (84.1%)	7 (15.9%)	0.429 (0.098, 2.093)	0.278
	T4	131	102 (77.9%)	29 (22.1%)	0.617 (0.171, 2.670)	0.489
Positive margins on specimen	Overall	199	154 (77.4%)	45 (22.6%)		0.788
	No	157	122 (77.7%)	35 (22.3%)	1.000	
	Yes	42	32 (76.2%)	10 (23.8%)	1.115 (0.488, 2.400)	0.788
Prophylactic antibiotic cover given to prevent fistula	Overall	199	154 (77.4%)	45 (22.6%)		0.230
	No	65	47 (72.3%)	18 (27.7%)	1.000	
	Yes	134	107 (79.9%)	27 (20.1%)	0.657 (0.334, 1.311)	0.230
Salivary bypass tube inserted	Overall	199	154 (77.4%)	45 (22.6%)		0.076
	No	175	132 (75.4%)	43 (24.6%)	1.000	
	Yes	24	22 (91.7%)	2 (8.3%)	0.338 (0.066, 1.108)	0.076
Reconstructive flap used	Overall	199	154 (77.4%)	45 (22.6%)		0.697
	No	137	107 (78.1%)	30 (21.9%)	1.000	
	Yes	62	47 (75.8%)	15 (24.2%)	1.150 (0.561, 2.293)	0.697

**Table 4:** Multiple-predictor binary logistic regression model for the outcome the occurrence of post-operative fistulae fitted using penalised maximum likelihood estimation

Predictor and category	Odds ratio	95% confidence limits		*p* value
		Lower	Upper	
Concurrent neck dissection (yes)	0.581	0.282	1.218	0.148
Post-operative Hb (g/dl)	0.853	0.691	1.039	0.115
Salivary bypass tube inserted (yes)	0.314	0.061	1.035	0.057

No co-variable was found to be significant in either the binary logistic regression model or multiple-predictor binary logistic regression model. In both models the closest co-variable to significance was the SBT with *p* values of 0.076 (odds ratio 0.338, 95% confidence intervals 0.066, 1.108) and 0.057 (odds ratio 0.314, 95% confidence interval 0.061–1.035), respectively.

**Fig. 1:**
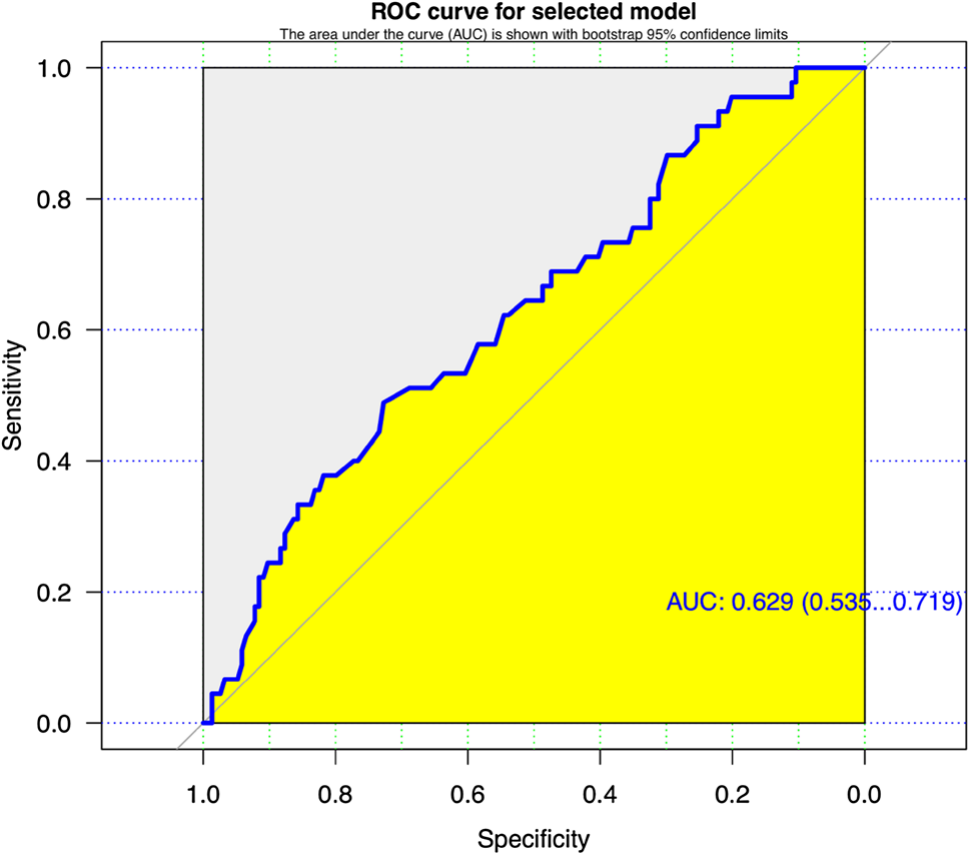
ROC curve with a value of 0.629 suggesting a moderate predictive performance

The binary logistic regression model must be tested to assess whether it is a good predictor for the occurrence of post-operative fistula and this is done by considering the ROC curve which is shown in Fig. 1. The area under the ROC curve (AUC) is 0.629 (95% CI 0.535–0.719) and is a measure of the models predictive performance. A value of 0.5 represents no predictive value and 1 perfect prediction. A value of 0.629 was achieved and this suggests the model has a moderate predictive performance.

## Discussion

Pharyngocutaneous fistulae are multifactorial in their origin [12] and there is a large amount of literature on the various causes. However, two systematic reviews have highlighted a lack of good quality research [8, 13]. Numerous potential risk factors have been identified but evidence to support which factors are significant is lacking due to small studies and the number of potential variables involved [8, 12].

Various factors have been associated with fistula development including suture material [14, 15], surgical experience [16] and the use of metronidazole [17, 18]. Patient co-morbidities are consistently identified as a significant risk factor [2, 8, 12, 19, 20]. A systematic review and meta-analysis by Paydarfar and Birkmeyer [8] found prior tracheostomy, pre-operative radiotherapy, a low post-operative haemoglobin and concurrent neck dissection to be associated with higher rates of PCF [8]. The effect of radiotherapy has been confirmed in a more recent systematic review and meta-analysis which failed to find a difference between chemoradiotherapy and radiotherapy groups; however, they did confirm flap reconstruction helps prevent PCF [13]. Prior radiotherapy is associated with an increased risk of PCF of 2.6 times [21]. In addition, fistulae seen in the salvage setting are larger than those seen following primary surgery [6].

There are numerous other suggested causes for PCF but they are disputed amongst groups. These include age [22, 23], positive histological margins [23–25], suture material [2, 12, 26], primary voice puncture [27–29], oral feeding [30], type of pharyngeal closure [19, 26, 31, 32]. The antibiotics given and their duration depending on the centre [33] but there is no clear evidence for any specific antibiotic regime [34] although clindamycin has been shown to increase complication rates [35].

Most fistulae will heal spontaneously but some take significantly longer, potentially requiring multidisciplinary team input and on occasion complex additional surgery [4]. Saliva coming into contact with the wound edges [36] and passing through the fistula defect are thought to be the cause of delayed closure [36, 37]. Saliva in contact with the wound leads to infection and micro-venous thrombosis resulting in tissue damage and destruction [12]. They delay post-operative oral feeding [4], increase inpatient hospital stay, morbidity and patient anxiety [2, 3, 8] and are associated with reduced quality of life [4]. Long-term complications include secondary dysphagia from fibrosis [12], and pharyngeal stenosis with potential requirements for further surgery to dilate strictures and or excise stenosed neopharynx with flap reconstruction [38].

Anecdotal evidence suggests SBT benefit patients [13, 39, 40] and reduce costs [10]. They were first described as a therapy for the prevention of PCF and stricture formation in 1978 [41]. The tubes are designed to divert solids and liquids which pass from the mouth to the oesophagus away from the anastomosis [42]. In addition, they are designed to prevent tube displacement into the throat and reflux of stomach contents and yet be large enough to allow oral feeding [42]. The SBT is made of non-adherent silicone and comes in various sizes up to 20 mm [36, 37]. It is shaped like a funnel and sits against the tongue base to channel saliva around the fistula and suture line theoretically promoting healing and fistula closure [36, 37]. Suturing the tubes to the tongue base [36] or through transcutaneous sutures [43] has been described to help secure them in place. Patients tolerate the tubes well although they are mainly used as a treatment for existing fistulae rather than in an attempt to prevent fistula development [44]. SBT’s are generally inserted and removed under a general anaesthetic [45] although local anaesthetic techniques have been described for insertion [46] and removal [9, 36]. Serious complications from pharyngeal SBT are rare but have been described as a direct result of their use. One patient developed peritonitis and went on to die following migration of the tube into the ileum [47] and arterio-esophageal fistulas have been described, one of which was fatal [48, 49]. However, they are generally safe with only mild complications such as discomfort and granulation tissue formation [43]. Although in theory, early oral intake may be possible, most surgeons prefer to feed patients via a nasogastric tube placed through the SBT in the early post-laryngectomy period [36].

An early study by Leon et al. [10] looked at 51 patient complications following pharyngolaryngectomy, neck dissection and pectoralis major flap for advanced cancer. They found no significant difference in complication rates between the SBT group and controls including PCF [10]. Their fistula rates were generally high at 47.4% in the SBT group and 60.9% in patients without a tube. Patient numbers were small, limiting the conclusions that could be drawn from this study, however, patients with SBTs spent less time in hospital, had a reduction in the severity of complications with less severe fistulas [10].

A univariate analysis by Bondi et al. [9] which examined the rate of PCF in matched patients with advanced tumour stages with and without a SBT found a fistula rate of 9% in the SBT group. Univariate analysis using the Chi-squared test showed a significant benefit of the salivary bypass tube in reducing fistula rates. No multivariate analysis was performed [9].

Punthakee et al. [11] performed a multicentre study with multivariate analysis on a sample 103 patients who had flap reconstruction, of which 54 patients had salivary bypass tubes inserted with a fistula rate of 7.4% [11]. Their univariate analysis showed a statistically significant association between lower rates of PCF and both flap reconstruction and salivary bypass tubes [11]. Unfortunately, their sample was not large enough to power the multivariate statistical analysis [11]. So an independent effect of SBT could not be confirmed.

The overall rate of PCF in our study of 22.6% is similar to the literature which quotes rates of between 3 and 65% [3–8, 26]. The falling rates of fistula following laryngectomy and lack of any statistically significant variable may be partly due to the introduction of IMRT and the MDT approach to surgery with better pre and post-operative care and identification of those at higher risk of fistula such as salvage cases receiving appropriate treatment strategies. In this study, no variables were found to be statistically significantly associated with PCF even those identified in previous studies such as radiotherapy.

The incidence of PCF in the group with a SBT was 8% (occurring in 2 out of the 24 patients). This compares well with other studies in the literature which records fistula rates in patients with SBT at between 0 and 21.8% [9–11, 37, 50]. We observed a difference in PCF rates in those patients who had a SBT versus those who did not (8.3 versus 24.6%) however, this failed to reach statistical significance.

There was a statistically significant association (on univariate analysis) between the use of salivary bypass tubes and free flap reconstruction. This may be explained by patient selection, as clinicians who have identified patients receiving a flap as high risk and used the SBT as added protection against a fistula and/or to help create a functioning pharynx and prevent stenosis in the flap reconstruction group.

## Limitations

Limitations of our study include its retrospective nature which may lead to bias in patients who receive the salivary bypass tube, with those who require more invasive surgery tending to be selected for SBT. However, it should be noted that when studying rare conditions retrospective analysis is usually the only study design option [51] and the results of well-designed retrospective studies usually agree with prospective studies [52]. Furthermore, there are potential observable and unobservable risk factors for PCF which are not included in this study and may be having a significant effect on results. It is likely that some surgeons reserve SBT for the patients perceived to be at highest risk of fistulae which may confound results. There was a statistically significant chance patients who received a flap reconstruction would receive a SBT (although this may be due to the low number of patients who received a SBT). This potential bias in patient selection cannot be completely excluded, however; we have attempted to address the issue of bias with the inclusion of multivariate analysis.

## Conclusions

In this study, which is the first to include sufficient numbers to allow multivariable analysis, we did not find an association between SBT and a reduction in PCF following total laryngectomy or pharyngolaryngectomy. We would recommend further research into salivary bypass tubes with a larger cohort of patients receiving a SBT or a prospective study design to increase understanding of their role in pharyngocutaneous fistulae. Despite the limitations of retrospective analysis our results suggest that any potential benefit is at most modest and as such the routine use of SBT cannot be recommended and surgeons must consider their use on a case by case basis.
